# Environmental risk assessment of the DvSSJ1 dsRNA and the IPD072Aa protein to non-target organisms

**DOI:** 10.1080/21645698.2021.1982348

**Published:** 2021-12-14

**Authors:** Chad J. Boeckman, Jennifer A. Anderson, Christopher Linderblood, Taylor Olson, Jason Roper, Kristine Sturtz, Carl Walker, Rachel Woods

**Affiliations:** Corteva Agriscience™, Johnston, IA USA

**Keywords:** DvSSJ1 dsRNA, IPD072AA, DP23211 maize, non-target organism, genetically modified, safety assessment, *Zea mays* L.

## Abstract

Event DP-Ø23211-2 (hereafter referred to as DP23211) maize expresses the DvSSJ1 double-stranded RNA (DvSSJ1 dsRNA) and the IPD072Aa protein, encoded by the *ipd072Aa* gene. DvSSJ1 dsRNA and the IPD072Aa protein each provide control of corn rootworms (*Diabrotica* spp.) when expressed in plants. As part of the environmental risk assessment (ERA), the potential hazard to non-target organisms (NTOs) exposed to the DvSSJ1 dsRNA and the IPD072Aa protein expressed in DP23211 maize was assessed. Worst-case estimated environmental concentrations (EECs) for different NTO functional groups (pollinators and pollen feeders, soil dwelling detritivores, predators and parasitoids, aquatic detritivores, insectivorous birds, and wild mammals) were calculated using worst-case assumptions. Several factors that reduce exposure to NTOs under more realistic environmental conditions were applied, when needed to provide more environmentally relevant EECs. Laboratory bioassays were conducted to assess the activity of DvSSJ1 dsRNA or the IPD072Aa protein against selected surrogate species, and margins of exposure (MOEs) were calculated by comparing the Tier I hazard study results to worst-case or refined EECs. Based on specificity and MOE values, DvSSJ1 dsRNA and the IPD072Aa protein expressed in DP23211 maize are not expected to be harmful to NTO populations at environmentally relevant concentrations.

## Introduction

Western corn rootworm (WCR; *Diabrotica virgifera virgifera* LeConte) is an insect pest of maize (*Zea mays* L.) that causes widespread economic damage and substantial crop loss. Resistance has developed in WCR populations to cultural control methods (crop rotation), some chemical insecticides, and some crystalline (cry) proteins derived from *Bacillus thuringiensis* (*Bt*) that are expressed in genetically modified (GM) maize.^1–3^ This presents a challenge for producers to successfully reduce crop injury by this agricultural pest. New *in-planta* modes of action against WCR are being developed^4–8^ to support a diverse management approach for controlling this difficult insect pest.

Event DP-Ø23211-2 (hereafter referred to as DP23211 maize) provides two new modes of action to control of corn rootworms (*Diabrotica* spp.) via expression of a DvSSJ1 double-stranded RNA (DvSSJ1 dsRNA) and the IPD072Aa protein.^9^ An environmental risk assessment was conducted, which considered among other things, the potential risks of the DvSSJ1 dsRNA and the IPD072Aa protein on non-target organisms (NTOs). The risk assessment framework described by the U.S. Environmental Protection Agency^10^ was employed and has been used for decades to assess GM crops derived from *Bacillus thuringiensis* (Bt). It is robust and suitable for assessing plants expressing non-Bt proteins^11^ and dsRNA.^5,12^

Problem formulation was used to guide the exposure and hazard assessments,^13–16^ and hypotheses for potential harm to NTO functional groups that provide beneficial ecosystem services were developed. The mode of action and the specificity of the gene products were considered to help focus the exposure and hazard assessment.^6,17,18^ The DvSSJ1 dsRNA is targeted to match a portion of the smooth septate junction protein 1 (*dvssj1*) gene from WCR. Smooth septate junctions (SSJs) are unique to invertebrate cells and are not found in vertebrates. When DvSSJ1 dsRNA in DP23211 maize is ingested by WCR, RNA interference (RNAi) down-regulates expression of the DvSSJ1 protein in the midgut, which disrupts the integrity of the SSJ, resulting in WCR mortality.^7,8^ The DvSSJ1 dsRNA has been shown to be highly specific, with activity limited to Coleoptera within the genus *Diabrotica* (Boeckman et al., in preparation). The IPD072Aa protein is encoded by the *ipd072* gene, which was derived from *Pseudomonas chlororaphis*, a soil bacterium with a history of safe use in agricultural applications.^11^ The IPD072Aa protein disrupts the midgut epithelial cell lining in WCR, resulting in mortality.^19^ Based on spectrum-of-activity testing, the insecticidal activity of the IPD072Aa protein is limited to the order Coleoptera.^6^

Relevant routes of exposure of the DvSSJ1 dsRNA and the IPD072Aa protein in DP23211 maize to different functional groups (pollinators and pollen feeders, soil dwelling detritivores, predators and parasitoids, aquatic detritivores, insectivorous birds, and wild mammals) were considered. Worst-case assumptions regarding exposure (e.g., maximum observed concentrations of the DvSSJ1 dsRNA and the IPD072Aa protein in relevant DP23211 maize tissues) were used to determine worst-case estimated environmental concentrations (EECs). Refined EECs were calculated using more realistic assumptions when there was an interest in understanding more environmentally relevant exposures. Laboratory bioassays were conducted to assess the activity of the DvSSJ1 dsRNA and the IPD072Aa protein against selected surrogate species.^15,20^ Margins of exposure (MOEs) were calculated by comparing these Tier I hazard study results to the worst-case or refined EECs. The MOE values were interpreted to assess potential risks associated with the cultivation of DP23211 maize to NTO populations.

## Methods

*DvSSJ1 dsRNA and IPD072Aa Protein Expression*: As described previously,^21^ DP23211 maize was planted during the 2018 growing season at eleven sites in the United States (including one site in Indiana, Minnesota, Nebraska, and Pennsylvania; two sites in Illinois and Texas; and three sites in Iowa), and one site in Ontario, Canada. Sites were selected to represent commercial maize growing regions of North America. Normal agronomic practices (irrigation, fertilization, herbicide and pesticide applications, etc.) were applied as needed and were applied uniformly to each entire trial area. Throughout the season, tissue samples were collected from DP23211 maize plants grown at six of these sites. QuantiGene analysis and a quantitative enzyme linked immunosorbent assay (ELISA) were used (as described in Supplemental Information) to determine the DvSSJ1 dsRNA and the IPD072Aa protein concentrations, respectively.

## Exposure Assessment

*Pollinators and Pollen Feeders*: For pollinators and pollen feeders, where the route of exposure is via consumption of DP23211 maize pollen, a worst-case scenario for exposure was assumed, based on consumption of only DP23211 maize pollen and maximum observed concentrations of DvSSJ1 dsRNA and the IPD072Aa protein in the pollen (2.02 × 10^−3^ ng DvSSJ1 dsRNA/mg and 1.3 ng IPD072Aa/mg dry weight; Table 1). Representative pollinators and pollen feeders considered in the exposure assessment included honey bee (larvae and adults), pollen-feeding non-target lepidopterans, and pollen-feeding coccinellids (Table 2). Honey bee larvae were assumed to consume 2 mg of maize pollen during larval development [^22^ Table 2]. Honey bee adults were assumed to consume 4.3 mg of maize pollen per day [^23^ Table 2].

**Table 1.** Concentration (dry weight) of the DvSSJ1 dsRNA and the IPD072Aa Protein DP23211 Maize Tissues

Tissue (Growth Stage)	ng DvSSJ1 dsRNA/mg			ng IPD072Aa Protein/mg		
	Mean	Maximum	Standard Deviation	Mean	Maximum	Standard Deviation
Root (V6)	5.13 × 10^−2^	9.44 × 10^−2^	2.08 × 10^−2^	25	60	25
Root (V9)	3.74 × 10^−2^	8.70 × 10^−2^	1.48 × 10^−2^	19	84	19
Root (R1)	2.91 × 10^−2^	5.85 × 10^−2^	1.00 × 10^−2^	21	51	21
Root (R4)	1.84 × 10^−2^	3.56 × 10^−2^	6.69 × 10^−3^	24	42	24
Root (R6)	1.15 × 10^−2^	3.57 × 10^−2^	8.31 × 10^−3^	31	72	31
Leaf (V9)	5.92 × 10^−2^	9.85 × 10^−2^	1.34 × 10^−2^	13	**39**	13
Leaf (R1)	4.97 × 10^−2^	9.67 × 10^−2^	1.79 × 10^−2^	**16**	33	16
Leaf (R4)	**6.46 × 10^−2^**	**1.13 × 10^−1^**	2.32 × 10^−2^	10	15	10
Leaf (R6)	1.32 × 10^−2^	3.31 × 10^−2^	1.05 × 10^−2^	1.6	10	1.6^a^
Pollen (R1)	9.87 × 10^−4^	**2.02 × 10^−3^**	3.09 × 10^−4^	0.65	**1.3**	0.65
Forage (R4)	1.90 × 10^−2^	5.65 × 10^−2^	1.07 × 10^−2^	16	28	16
Whole Plant (R1)	**2.19 × 10^−2^**	3.59 × 10^−2^	5.10 × 10^−3^	7.9	14	7.9
Whole Plant (R6)	1.08 × 10^−2^	**2.99 × 10^−2^**	5.40 × 10^−3^	**11**	**24**	11
Grain (R6)	4.13 × 10^−3^	**1.09 × 10^−2^**	2.36 × 10^−3^	2.1	**4.8**	2.1

Mean and/or maximum values determined to be relevant for the exposure assessment are highlighted in bold text.

^a^Some, but not all, sample results were below the LLOQ. A value equal to half the LLOQ value was assigned to those samples to calculate the mean and standard deviation.

**Table 2.** Worst-case and Refined Estimated Environmental Concentrations (EEC) for Functional Groups of Non-Target Organisms Exposed to DvSSJ1 dsRNA and the IPD072Aa Protein from DP23211 Maize

Functional Groups	Route of Exposure	Assumptions		Worst-case and Refined EEC	
		Worst-case	Refined	DvSSJ1 dsRNA	IPD072Aa Protein
**Pollinators and Pollen Feeders**	Consumption of DP23211 maize pollen	Honeybee larvae Consume 2 mg of pollen during larval development^1^ Exposed to the maximum pollen concentration	No refinement to worst-case EEC considered	Worst-case EEC = 4.04 × 10^−3^ ng/larvae Refined EEC = NA^2^	Worst-case EEC = 2.6 ng/larvae Refined EEC = NA
		Honeybee adults Consume 4.3 mg of pollen per day^1^ Exposed to the maximum pollen concentration	No refinement to worst-case EEC considered	Worst-case EEC = 8.69 × 10^−3^ ng/bee Refined EEC = NA	Worst-case EEC = 5.59 ng/bee Refined EEC = NA
		Non-target Lepidoptera Exposed to the maximum pollen concentration	No refinement to worst-case EEC considered	Worst-case EEC = 2.02 × 10^−3^ ng/mg Refined EEC = NA	Worst-case EEC = 1.3 ng/mg Refined EEC = NA
		Pollen feeding coccinellids Exposed to the maximum pollen concentration	No refinement to worst-case EEC considered	Worst-case EEC = 2.02 × 10^−3^ ng/mg Refined EEC = NA	Worst-case EEC = 1.3 ng/mg Refined EEC = NA
**Soil-Dwelling Detritivores**	Consumption of senescent DP23211 maize tissue	Exposed to the maximum concentration in senescent (R6) whole plant tissue	No refinement to worst-case EEC considered	Worst-case EEC = 2.99 × 10^−2^ ng/mg Refined EEC = NA	Worst-case EEC = 24 ng/mg Refined EEC = NA
**Aquatic Detritivores**	Consumption of DP23211 maize tissue	EPA standard pond model ^3^ Exposed to the highest mean whole plant concentration across any growth stage	No refinement to worst-case EEC considered	Worst-case EEC = 2.46 × 10^−4^ mg/ml Refined EEC = NA	Worst-case EEC = 0.124 mg/ml Refined EEC = NA
**Herbivores**	Consumption of DP23211 maize tissue	Herbivorous Coccinellids Exposed to the maximum concentration in above-ground tissue (highest expressing tissue across the growing season)	Consume mean concentration in above-ground tissue (highest mean concentration across the growing season)	Worst-case EEC = 0.113 ng/mg Refined EEC = 0.0646 ng/mg	Worst-case EEC = 39 ng/mg Refined EEC = 16 ng/mg
**Predators and Parasitoids**	Consumption of prey that has consumed DP23211 maize tissue	Prey consumes the maximum concentration in above-ground tissue (highest expressing tissue across the growing season) No degradation of the dsRNA or protein in prey.	Prey consume the mean concentration in above-ground tissue (highest mean concentration across the growing season) No degradation of the dsRNA or protein in prey.	Worst-case EEC = 0.113 ng/mg Refined EEC = 0.0646 ng/mg	Worst-case EEC = 39 ng/mg Refined EEC = 16 ng/mg
**Insectivorous Birds**	Consumption of prey that has consumed DP23211 maize tissue	Prey consumes the maximum concentration in above-ground tissue (highest expressing tissue across the growing season) No degradation of the dsRNA or protein in prey.	Prey consume the mean concentration in above-ground tissue (highest mean concentration across the growing season) No degradation of the dsRNA or protein in prey.	Worst-case EEC = 0.113 ng/mg Refined EEC = 0.0646 ng/mg	Worst-case EEC = 39 ng/mg Refined EEC = 16 ng/mg
**Granivorous Mammals**	Consumption of DP23211 maize grain	DDD *^4^* = FIR/BW * C Exposed to the maximum concentration in grain (R6). 73% of diet consists of maize grain	No refinement to worst-case EEC considered	.Worst-case EEC = 0.0026 mg/kg body weight Refined EEC = NA	Worst-case EEC = 1.156 mg/kg body weight Refined EEC = NA

^1^Babendreier et al., 2004; Crailsheim et al., 1992.

^2^NA; No refinement to worst-case EEC considered.

^3^as described previously (Carstens et al., 2016). All of the above ground maize tissue from a 10-hectare (ha) field was assumed to be deposited in a 1-ha pond (2 meters deep; equivalent to 20,000,000 L of water); maize planting density was assumed to be 75,000 plants/ha (equivalent to 750,000 plants); and one maize plant was assumed to weigh 0.3 kg (dry weight; which is equivalent to 225,000 kg of maize tissue).

^4^DDD = FIR/BW * Concentration (Crocker et al., 2002; Raybould et al., 2007); the worst-case FIR/BW ratio for seed-eating rodents is 0.33 for the harvest mouse (*Micromys minutus*) (Raybould et al., 2007). 73% of the mammal’s diet consists of maize grain (Raybould et al., 2007).

*Soil dwelling detritivores*: For soil dwelling detritivores, the route of exposure to the DvSSJ1 dsRNA and the IPD072Aa protein is via consumption of senescent DP23211 maize tissues that are deposited or incorporated into the soil (Table 2). The worst-case EEC for soil-dwelling organisms was calculated using the maximum concentration of the DvSSJ1 dsRNA and the IPD072Aa protein in senescent (R6) whole plant tissue (2.99 × 10^−2^ ng/mg and 24 ng/mg, respectively; Table 1).

*Aquatic detritivores*: For aquatic detritivores, the route of exposure to the DvSSJ1 dsRNA and the IPD072Aa protein is via exposure to DP23211 maize tissues that enter water (Table 2). The worst-case EEC for aquatic detritivores was estimated using the EPA standard agricultural field-farm pond model. Worst-case assumptions were applied to the model, as reported previously.^24^ Briefly, all of the above-ground maize tissue from a 10-hectare (ha) field was assumed to be deposited in a 1-ha pond (2 m deep; equivalent to 20,000,000 L of water); maize planting density was assumed to be 75,000 plants/ha; and one maize plant was assumed to weigh 0.3 kg (dry weight). The mean concentration of the DvSSJ1 dsRNA and the IPD072Aa protein concentration in whole plant tissues was determined to be highest at the R1 growth stage for DvSSJ1 dsRNA (2.19 × 10^−2^ ng/mg; Table 1) and at the R6 growth stage for IPD072Aa protein (11 ng/mg; Table 1); therefore, these concentrations were used to calculate the worst-case EECs. Mean concentrations were considered in this case because all of the maize tissue from the entire 10-ha field was assumed to enter the water.

*Herbivores*: Most insects that consume maize tissue directly are considered agricultural pests; however, some beneficial non-target organisms may consume maize tissues [for example, coccinellids;^25^ Table 2]. For the worst-case EEC, we assumed that an herbivore was exposed to the highest maximum concentration from any above-ground plant tissue and from any growth stage (0.113 ng DvSSJ1 dsRNA/mg from R4 leaf and 39 ng IPD072Aa protein/mg from V9 leaf; Table 1). For a refined EEC, we assumed that an herbivore was exposed to the highest mean concentration from any above-ground plant tissue and from any growth stage (0.0646 ng DvSSJ1 dsRNA/mg from R4 leaf and 16 ng IPD072Aa protein/mg from R1 leaf; Table 1).

*Predators, Parasitoids, and Insectivorous Birds*: For insect predators or parasitoids, the route of exposure to the DvSSJ1 dsRNA and the IPD072Aa protein is via consumption of insect prey that has previously consumed tissue from a DP23211 maize plant (Table 2). Similar to predatory insects, some wild birds may consume insect prey that has consumed DP23211 maize tissue (Table 2). For the worst-case EECs for predators, parasitoids, and insectivorous birds we assumed that there was no degradation of the DvSSJ1 dsRNA or the IPD072Aa protein in prey, and 100% of the DvSSJ1 dsRNA or the IPD072Aa protein was transferred from DP23211 maize tissue to prey and then on to the predator, parasitoid, or insectivorous bird. Because the EEC for predators, parasitoids, or insectivorous birds does not factor any loss of the DvSSJ1 dsRNA or the IPD072Aa protein during tri-trophic transfer, the worst-case and refined EECs for predators or predator, parasitoid, or insectivorous bird are the same as the worst-case and refined EECs for herbivores that consume the DP23211 maize tissue directly (Table 2).

*Granivorous Mammals*: For granivorous mammals, the route of exposure to the DvSSJ1 dsRNA and the IPD072Aa protein is via consumption of DP23211 maize grain (Table 2). For the worst-case EEC, we used the maximum concentration of the DvSSJ1 dsRNA and the IPD072Aa protein in DP23211 grain (1.09 × 10^−2^ ng/mg and 4.8 ng/mg, respectively; Table 1) and assumed that 73% of the diet contained maize grain.^26^ A daily dietary dose (DDD) for wild rodents accounts for food intake rate (FIR) and body weight (BW) and was calculated using the approach previously described by ^26,27^ and The worst-case FIR/BW ratio for seed-eating rodents previously determined for the harvest mouse (*Micromys minutus*^26^) was used to calculate the worst-case EECs (Table 2).

## Hazard Assessment

All hazard studies were conducted in compliance with Good Laboratory Practice regulations as provided in EPA 40 CFR part 160.^28^ For IPD072Aa protein bioassays, the IPD072Aa protein was expressed in *E. coli*, lyophilized, and characterized as described previously.^6^ For DvSSJ1 dsRNA bioassays, an RNA oligonucleotide DvSSJ1_210 dsRNA was produced by Genolution Inc. (Seoul, Korea) using a proprietary method. The equivalence between the plant expressed and *E. coli* produced IPD072Aa protein was established. Similarly, the equivalence between the plant expressed DvSSJ1 dsRNA and the DvSSJ1_210 dsRNA was confirmed (see supplemental materials for further description of both test substances). DvSSJ1 dsRNA and IPD072Aa bioassays were conducted in separate experiments, based on the lack of biologically meaningful synergism observed in an organism sensitive to both DvSSJ1 dsRNA and IPD072Aa protein. To assess for synergism between the two actives, median lethal concentrations were first established using multiple 14-d bioassays with each active alone (not shown). Subsequently, three additional 14-d bioassays were conducted using mixtures of the two actives at each respective LC_10_, LC_20_, LC_30_, LC_40_ and LC_50_ concentrations. Additional treatments included a negative control and each active alone at the established LC_50_ value to serve as a control to understand the consistency of performance of multiple batches of WCR. The independent model of combined action^29^ was used to estimate the potency of the mixtures. The independent action model is appropriate to assess the combined potency given the different modes of action between DvSSJ1 dsRNA and IPD072Aa^30,31^; however,^32^have suggested the independent model of combined action may underestimate toxicity of mixtures (and therefore falsely conclude mixtures are acting synergistically) perhaps due to generalized physiological effects within organisms exposed to combinations of stressors. Table 3 shows the observed mortality of WCR associated with each bioassay and each treatment. Mortality in each bioassay was first corrected for the natural mortality observed in the negative control. A generalized linear model assuming a binomial distribution and a logit link function was used to fit the adjusted mortality data as responding to the treatment. Mortality percentages for each treatment across the three bioassays were then estimated with 95% confidence intervals. The 95% confidence intervals for mortality associated with each of the individual actives alone at the previously estimated LC_50_ included 50% mortality indicating the performance of WCR used to assess for synergism between the two actives was sufficiently similar to the previous bioassays used to estimate the LC_50_ values. As shown in Table 3, observed WCR mortality with mixtures of the two actives at the LC_10_ and LC_20_ was consistent with the predicted mortality from the independent model of combined action. Observed mortality of WCR at the LC_30_, LC_40_ and LC_50_ mixtures exceeded the model predicted mortality. Based on regulatory guidance documents a five-fold increase in potency was considered as a threshold beyond which additional work would be necessary to further characterize the potency of the combined mixtures.^31^ As several treatments were showing greater than model predicted mortality, the independent model of combined activity was again used to derive hypothetically expected mortality of a mixture acting synergistically at a two-fold level (Table 3). Given all mixtures except the combination of actives at the LC_50_s (where 100% mortality was observed) are showing potency less than a two-fold level and given the independent model of combined activity may underestimate potency of mixtures, the threshold for additional mixture characterization was not met and therefore hazard studies conducted with the individual test substances are informative for the risk assessment.

**Table 3.** Results of Mixture Testing with Western Corn Rootworm Fed DvSSJ1 DsRNA and IPD072Aa Protein

Treatment	Bioassay	Corrected Mortality (%)	Estimated % Mortality (95% Confidence Interval)	Model Predicted Mortality (%)	Model Predicted Mortality (%) Assuming 2-fold Synergism
**Negative Control Diet**	1	0	0	-	-
	2	0			
	3	0			
**LC_50_ of IPD072Aa**	1	30.4	47.3 (34.3 – 60.7)	-	-
	2	51.9			
	3	65.5			
**LC_50_ of DvSSJ1**	1	50.4	58.2 (45.0 – 70.3)	-	-
	2	79.3			
	3	34.0			
**LC_10_ of each active**	1	13.0	20.5 (11.9 – 33.0)	19	51.5
	2	25.0			
	3	23.0			
**LC_20_ of each active**	1	30.4	38.0 (26.2 – 51.4)	36	73.5
	2	53.6			
	3	22.3			
**LC_30_ of each active**	1	54.9	71.3 (58.6 – 81.4)	51	84.9
	2	83.3			
	3	71.6			
**LC_40_ of each active**	1	76.6	80.7 (68.1 – 89.1)	64	91.3
	2	86.7			
	3	75.0			
**LC_50_ of each active**	1	100	100	75	95.1
	2	100			
	3	100			

In all bioassays, the DvSSJ1 dsRNA or IPD072Aa protein concentrations in diets were characterized to confirm stability, homogeneity in diet, and concentration. Depending on the bioassay, fresh diet was prepared and replaced daily, every 3–4 d, or as needed to maintain the DvSSJ1 dsRNA or the IPD072Aa protein above 70% of the target concentration. Appropriate positive controls were included with each study to verify exposure of each organism to the DvSSJ1 dsRNA or the IPD072Aa protein in the diet. A negative control, consisting of diet without either the DvSSJ1 dsRNA or the IPD072Aa protein, was included to assess bioassay performance. In general, acceptability criteria required ≤20% mortality in the negative control over a 7-d duration for IPD072Aa or 14-d duration for DvSSJ1 dsRNA, and at least 80% mortality in the positive control. To demonstrate the bioactivity of the DvSSJ1 dsRNA or the IPD072Aa protein in diet, a portion of the diet fed to each surrogate species was incorporated into diet and fed to WCR.

Surrogate species were selected based on the exposure assessment, understanding of the specificity of DvSSJ1 dsRNA or the IPD072Aa protein, [^6^ Boeckman et al., in preparation], as well as practical considerations (e.g., availability of laboratory-reared insects and established, reproducible, and robust methods). Surrogate species assessed included *Apis mellifera* (honey bee larvae and adults), *Folsomia candida* (springtail), *Chrysoperla rufilabris* (green lacewing), *Coleomegilla maculata* (pink spotted lady beetle), *Hippodamia convergens* (convergent ladybird beetle), *Dalotia coriaria* (rove beetle), *Pediobius foveolatus* (parasitic hymenoptera), *Colinus virginianus* (Northern bobwhite quail), and *Mus musculus* (mouse), which represent functional groups of pollinators and pollen feeders, soil-dwelling detritivores, predators and parasitoids, insectivorous birds, and granivorous mammals (Tables 4 and Tables 5). Insect bioassays were conducted in small environmental chambers, with temperatures ranging from 21°C to 30°C depending on the insect of interest. The light regime was maintained at either continuously dark or with a 16 L:8D photoperiod, and relative humidity was generally maintained above 65%. Most of the bioassays were initiated with neonates, less than 24-h old unless otherwise noted (Tables 4 and Tables 5).

**Table 4.** Early Tier Laboratory Study Results and Margins of Exposure (MOEs) for the DvSSJ1 dsRNA on Representative Non-Target Organisms

	Species (Common Name)	Duration of Study and Concentration	Endpoints Assessed	Results ^1^	Margin of Exposure (MOE) ^2^
**Pollinators and Pollen Feeders**	*Apis mellifera* (Honeybee larvae)	22-d: Diet containing 4.0 ng DvSSJ1 dsRNA/larvae	Larval survival, pupal survival, adult emergence, or adult weight at emergence	No adverse effects on larval survival, pupal survival, adult emergence, or adult weight at emergence. The survival NOED is 4.0 ng DvSSJ1 dsRNA/larvae	MOE = 990X based on a worst-case EEC.
	*Apis mellifera* (Honeybee adults)	14-d: Diet containing a mean daily dose of 26 ng DvSSJ1 dsRNA/bee/day	Survival, adult weight	No adverse effects on survival or adult weight were observed. The survival NOEDD is 26 ng DvSSJ1 dsRNA/bee/day	MOE = 2,993X based on a worst-case EEC.
	Non-target lepidopterans	Tier I hazard studies on non-target lepidopterans were not conducted based on negligible potential for exposure and lack of effects of DvSSJ1 dsRNA observed on Lepidoptera as part of the spectrum of activity assessment (Boeckman et al., submitted). The LC_50_ of the DvSSJ1 dsRNA for the most sensitive target pest (*D. virgifera virgifera*) is 0.036 ng DvSSJ1 dsRNA/mg (Boeckman et al., in preparation)			MOE = 18X (based on a worst-case EEC for non-target Lepidoptera and the LC_50_ of *D. virgifera virgifera*).
	Pollen feeding coccinellids	*Coleomegilla maculata* and *Hippodamia convergens* bioassays were conducted (see details of Tier 1 hazard studies summarized in the predator and parasitoid functional group). The predator and parasitoid route of exposure was determined to be higher than the Pollinators and Pollen Feeders route of exposure for non-target coccinellids. The LC_50_ of the DvSSJ1 dsRNA for the most sensitive target pest (*D. virgifera virgifera*) is 0.036 ng DvSSJ1 dsRNA/mg (Boeckman et al., in preparation), which was used to estimate the MOE for pollen feeding coccinellids.			MOE = 18X (based on a worst-case EEC for pollen feeding coccinellids and the LC_50_ of *D. virgifera virgifera*).
**Soil-Dwelling Detritivores**	*Folsomia candida* (Springtail)	28-d: Diet containing 1 ng DvSSJ1 dsRNA/mg diet	Survival, reproduction	No adverse effect on survival or reproduction were observed. The survival NOEC is 1 ng DvSSJ1 dsRNA/mg diet	MOE = 33X based on a worst-case EEC.
**Aquatic Detritivores**	Aquatic detritivores	Tier I hazard studies on non-target aquatic organisms for the IPD072Aa protein were not conducted based on negligible potential for exposure. The LC_50_ of the DvSSJ1 dsRNA for the most sensitive target pest (*D. virgifera virgifera*) is 0.036 ng DvSSJ1 dsRNA/mg (Boeckman et al., submitted), which was used to estimate the MOE for non-target aquatic organisms.			MOE = 146X (based on a worst-case EEC for non-target aquatic organisms and the LC_50_ of *D. virgifera virgifera*).
**Predators and Parasitoids**	*Chrysoperla rufilabris* (Green lacewing)	21-d: 1 ng DvSSJ1 dsRNA/mg	Survival, pupation	No adverse effects on survival or pupation were observed The survival NOEC is 1 ng DvSSJ1 dsRNA/mg diet	MOE = 9X based on a worst-case EEC and 15X based on a refined EEC.
	*Coleomegilla maculata* (Pink spotted lady beetle)	28-d: 1 ng DvSSJ1 dsRNA/mg	Survival, weight, days to adult emergence	No adverse effects on survival, weight, or number of days to adult emergence were observed. The survival NOEC is 1 ng DvSSJ1 dsRNA/mg diet	MOE = 9X based on a worst-case EEC and 15X based on a refined EEC.
	*Hippodamia convergens* (Convergent ladybird beetle)	28-d: 1 ng DvSSJ1 dsRNA/mg	Survival, weight, days to adult emergence	No adverse effects on survival, weight, or number of days to adult emergence were observed. The survival NOEC is 1 ng DvSSJ1 dsRNA/mg diet	MOE = 9X based on a worst-case EEC and 15X based on a refined EEC.
	*Dalotia coriari* (Rove beetle)	14-d: 1 ng DvSSJ1 dsRNA/mg	Survival	No adverse effects on survival were observed. The survival NOEC is 1 ng DvSSJ1 dsRNA/mg diet	MOE = 9X based on a worst-case EEC and 15X based on a refined EEC.
	*Pediobius foveolatus* (parasitic hymenoptera)	7-d: 1 µg DvSSJ1 dsRNA/ml	Survival	No adverse effect on survival were observed. The survival NOEC is 1 µg DvSSJ1 dsRNA/ml	MOE = 9X based on a worst-case EEC and 15X based on a refined EEC.
**Insectivorous Birds**	*Colinus virginianus* (Northern bobwhite quail)	14-d: 105 mg DvSSJ1 dsRNA/kg body weight	Mortality, body weight, abnormal behavior, signs of toxicity	No adverse effects on survival, weight, abnormal behavior or signs of toxicity were observed. The NOEL and the LD_50_ are >105 mg DvSSJ1 dsRNA/kg body weight	MOE = 929X based on a worst-case EEC and 1,625X based on a refined EEC.
**Granivorous Mammals**	*Mus musculus* (Mouse)	Based on the low potential for exposure to non-target granivorous mammals, Tier I hazard assessment was not conducted for the DvSSJ1 dsRNA. There are barriers to uptake of dsRNA that likely limit exposure of mammals to the DvSSJ1 dsRNA			

^1^Note: median lethal concentration (LD_50_), no observed effect concentration (NOEC), no observed effect dose (NOED), or no observed effect dietary-dose (NOEDD)

^2^All MOEs are rounded to the nearest whole number and are calculated based on tissue dry weight (DW). The dry weight concentrations are considered high estimates, since in reality NTOs would be exposed to levels comparable to fresh weight levels (US-EPA, 2017). Insect bioassays may be reported based on wet weight or dry weight concentrations.

**Table 5.** Early Tier Laboratory Study Results and Margins of Exposure (MOEs) for the IPD072Aa Protein on Representative Non-Target Organisms

	Species (Common Name)	Concentration	Endpoints Assessed	Results ^1^	Margin of Exposure (MOE) ^2^
**Pollinators and Pollen Feeders**	*Apis mellifera* (Honeybee larvae)	22-d: Diet containing 100 or 200 ng IPD072Aa protein/larvae	Larval survival, pupal survival, adult emergence, or adult weight at emergence	No adverse effects on larval survival, pupal survival, adult emergence, or adult weight at emergence were observed. The survival NOED is 200 ng IPD072Aa protein/larvae	MOE = 77X based on a worst-case EEC.
	*Apis mellifera* (Honeybee adults)	10-d: Diet containing a mean daily dose of 1,300 ng IPD072Aa protein/bee/day	Survival, adult weight	No adverse effects on survival or adult weight were observed. The survival NOEDD is 1,300 ng IPD072Aa protein/bee/day	MOE = 233X based on a worst-case EEC.
	Non-target lepidopterans	Tier I hazard studies on non-target lepidopterans for the IPD072Aa protein were not conducted based on negligible potential for exposure and lack of effects observed in the spectrum of activity assessment (Boeckman et al., 2019). The LC_50_ of the IPD072Aa protein for the most sensitive target pest (*D. virgifera virgifera*) is 26 ng/mg (Boeckman et al., 2019)			MOE = 20X (based on a worst-case EEC for non-target Lepidoptera and the LC_50_ of *D. virgifera virgifera*).
	Pollen feeding coccinellids	*Coleomegilla maculata* and *Hippodamia convergens* bioassays were conducted (see details of Tier 1 hazard studies summarized in the predator and parasitoid functional group). The predator and parasitoid route of exposure was determined to be higher than the Pollinators and Pollen Feeders route of exposure for non-target coccinellids. The LC_50_ of the IPD072Aa protein for the most sensitive target pest (*D. virgifera virgifera*) is 26 ng/mg (Boeckman et al., 2019), which was used to estimate the MOE for pollen feeding coccinellids.			MOE = 20X (based on a worst-case EEC for pollen feeding coccinellids and the LC_50_ of *D. virgifera virgifera*).
**Soil-Dwelling Detritivores**	*Folsomia candida* (Springtail)	28-d: Diet containing 500 ng IPD072Aa protein/mg	Survival, reproduction	No biologically relevant adverse effect on springtail survival or reproduction observed ^3^. The survival NOEC is 500 ng IPD072Aa protein/mg diet	MOE = 21X based on a worst-case EEC.
**Aquatic Detritivores**	Aquatic Detritivore	Tier I hazard studies on non-target aquatic organisms for the IPD072Aa protein were not conducted based on negligible potential for exposure. The LC_50_ of the IPD072Aa protein for the most sensitive target pest (*D. virgifera virgifera*) is 26 ng/mg (Boeckman et al., 2019), which was used to estimate the MOE for non-target aquatic organisms.			MOE = 210X (based on a worst-case EEC for non-target aquatic organisms and the LC_50_ of *D. virgifera virgifera*).
**Predators and Parasitoids**	*Chrysoperla rufilabris* (Green lacewing)	21-d: Diet containing 500 ng IPD072Aa protein/mg	Survival, pupation	No adverse effects on survival or pupation of green lacewing were observed The survival NOEC is 500 ng IPD072Aa protein/mg diet	MOE = 13X based on a worst-case EEC and 31X based on a refined EEC.
	*Coleomegilla maculata* (Pink spotted lady beetle)	28-d: Diet containing 100, 500, and 1000 ng IPD072Aa protein/mg diet	Survival, weight, days to adult emergence	No adverse effects on survival, weight, or number of days to adult emergence were observed at 100 ng IPD072Aa protein/mg diet ^4^. The survival NOEC is 100 ng IPD072Aa protein/mg diet	MOE = 3X based on a worst-case EEC and 6X based on a refined EEC.
	*Hippodamia convergens* (Convergent ladybird beetle)	28-d: Diet containing 100, 500, and 1000 ng IPD072Aa protein/mg diet	Survival, weight, days to adult emergence	Exposure to 100 ng and 500 ng IPD072Aa protein/mg diet had no adverse effects on survival ^4^. The survival NOEC is 500 ng IPD072Aa protein/mg diet	MOE = 13X based on a worst-case EEC and 31X based on a refined EEC.
	*Dalotia coriari* (Rove beetle)	7-d: Diet containing 100, 500, and 1000 ng IPD072Aa protein/mg diet	Survival	No adverse effects on survival were observed. The survival NOEC is 1000 ng IPD072Aa protein/mg diet	MOE = 26X based on a worst-case EEC and 63X based on a refined EEC.
	*Pediobius foveolatus* (parasitic hymenoptera)	7-d: Diet containing 100, 500, and 1000 µg IPD072Aa protein/ml diet	Survival	No adverse effect on survival were observed. The survival NOEC is 1000 µg IPD072Aa protein/ml diet	MOE = 26X based on a worst-case EEC and 63X based on a refined EEC.
**Insectivorous Birds**	*Colinus virginianus* (Northern bobwhite quail)	14-d limit dose: 2000 mg IPD072Aa protein/kg body weight	Mortality, body weight, abnormal behavior, signs of toxicity	No mortality, abnormal behavior or signs of toxicity were observed. The NOEC and the LD_50_ are >2000 mg IPD072Aa protein/kg body weight.	MOE = 51X based on a worst-case EEC and 125X based on a refined EEC.
**Granivorous Mammals**	*Mus musculus* (Mouse)	14-d: 2000 mg IPD072Aa protein/kg body weight	Mortality, evidence of acute oral toxicity (based on evaluation of body weight, clinical signs, and gross pathology).	No mortality or other evidence of acute oral toxicity was observed, based on evaluation of body weight, clinical signs, and gross pathology. The LD_50_ is >2000 mg IPD072Aa protein/kg body weight.	MOE = 1730X based on a worst-case EEC.

^1^Note: median lethal concentration (LD_50_), no observed effect concentration (NOEC), no observed effect dose (NOED), or no observed effect dietary-dose (NOEDD).

^2^All MOEs are rounded to the nearest whole number and are calculated based on tissue dry weight (DW). The dry weight concentrations are considered high estimates, since in reality NTOs would be exposed to levels comparable to fresh weight levels (US-EPA, 2017). Insect bioassays may be reported based on wet weight or dry weight concentrations.

^3^A statistically significant difference was observed for the sub-lethal endpoint pupation (mean number of offspring per jar), however this was not biologically meaningful based on acceptability guidelines for collembolan reproduction established by OECD, as well as the overlapping range of offspring observed across treatments.

^4^mortality and sublethal effects were observed at the higher concentrations (Supplemental Table 2).

*Honey bee larvae*: Honey bee larvae (*Apis mellifera*) were exposed to diet containing 4 ng DvSSJ1 dsRNA/larvae (Table 4). In a separate study, honey bee larvae were exposed to diet containing IPD072Aa protein (100 and 200 ng IPD072Aa protein/larvae; Table 5). Bioassays were 22-d in duration and followed OECD Guidance Document No. 239. In each study, there were 36 replicates per treatment, and freshly prepared diet containing one of the following treatments (DvSSJ1 dsRNA or the IPD072Aa protein treatments, negative control, or positive control, which consisted of dimethoate, a known honey bee toxicant) was provided on four occasions prior to pupation. The endpoints that were assessed included larval and pupal survival, adult emergence, and adult weight at emergence.

*Honey bee adults*: Honey bee adults (*Apis mellifera*; ≤2-d old emerged) were exposed to diet containing a mean daily dose of 26 ng DvSSJ1 dsRNA/bee/day for 14 d (Table 4). In a separate study, honey bee adults were exposed to diet containing a mean daily dose of 1,300 ng IPD072Aa protein/bee/day for 10 d (Table 5). Bioassays followed OECD Guidance Document No. 245. In each study, there were 30 replicates per treatment, and freshly prepared diet containing one of the following treatments (DvSSJ1 dsRNA or the IPD072Aa protein, negative control, or the positive control dimethoate) was provided daily. The endpoints that were assessed included survival and adult weight.

Springtail (*Folsomia candida*) adults were exposed to diet containing 1 ng DvSSJ1 dsRNA/mg diet for 28 d (Table 4). In a separate study, springtail adults were exposed to diet containing 500 ng IPD072Aa protein/mg diet for 28 d (Table 5). Springtail were housed in small wide-mouth glass jars (8 replicate jars containing a target of 10 individuals each) and were provided freshly prepared diet containing one of the following treatments (DvSSJ1 dsRNA or the IPD072Aa protein, negative control, or positive control, which consisted of teflubenzuron) daily. The endpoints that were assessed included survival and reproduction.

Green lacewing (*Chrysoperla rufilabris*) neonates were exposed to diet containing 1 ng DvSSJ1 dsRNA/mg diet for 21 d (Table 4). In a separate study, green lacewing neonates were exposed to diet containing 500 ng IPD072Aa per mg diet for 21 d (Table 5). Green lacewings were housed in 30 mL plastic cups (40 replicates per treatment) and were provided freshly prepared diet containing one of the following treatments (DvSSJ1 dsRNA or the IPD072Aa protein, negative control, or positive control, which consisted of cryolite) daily. The endpoints that were assessed included survival and pupation.

Spotted lady beetle (*Coleomegilla maculata*) neonates were exposed to a diet containing 1 ng DvSSJ1 dsRNA/mg diet (Table 4). In a separate study, *C. maculata* larvae were exposed to diets containing 100, 500, or 1000 ng IPD072Aa protein/mg diet (Table 5). The study duration was 28 d or until adult emergence. *C. maculata* larvae were housed individually in Petri dishes (30 replicates per treatment) and were provided freshly prepared diet containing one of the following treatments (DvSSJ1 dsRNA or the IPD072Aa protein treatments, negative control, or positive control, which consisted of cryolite) every 3–4 d. The endpoints that were assessed included survival, weight, and number of days to adult emergence.

Convergent lady beetle (*Hippodamia convergens*) neonates were exposed to a diet containing 1 ng DvSSJ1 dsRNA/mg diet (Table 4). In a separate study, *H. convergens* neonates were exposed to diets containing 100, 500, or 1000 ng IPD072Aa protein/mg (Table 5). The study duration was 28 d or until adult emergence. *H. convergens* were housed individually in Petri dishes (30 replicates per treatment) and were provided freshly prepared diet containing one of the following treatments (DvSSJ1 dsRNA or the IPD072Aa protein treatments, negative control, or positive control, which consisted of boric acid) every 3–4 d. The endpoints that were assessed included survival, weight, and number of days to adult emergence.

Rove beetle (*Dalotia coriaria*) adults were exposed to a diet containing 1 ng DvSSJ1 dsRNA/mg diet (Table 4). In a separate study, *D. coriaria* adults were exposed to diets containing 100, 500, or 1000 ng IPD072Aa protein/mg (Table 5). The study durations were 14 d and 7 d, respectively. *D. coriaria* were housed in 30 mL plastic cups (1 individual per cup), for a total of 30 individuals per treatment and were provided freshly prepared diet containing one of the following treatments (DvSSJ1 dsRNA or the IPD072Aa protein treatments, negative control, or positive control, which consisted of boric acid) daily. The endpoint assessed was survival.

Parasitoid wasp (*Pediobius foveolatus*) adults were exposed to a 30% sucrose diet containing 1 ng DvSSJ1 dsRNA/mg diet (Table 4). In a separate study, *P. foveolatus* adults were exposed to a 30% sucrose diet containing 100, 500, or 1000 µg IPD072Aa protein/ml diet (Table 5). The study duration was 14 d for DvSSJ1 and 7 d for IPD072Aa protein. *P. foveolatus* were housed in 30 mL plastic cups (30 replicates per treatment) and were provided freshly prepared diet containing one of the following treatments (DvSSJ1 dsRNA or the IPD072Aa protein treatments, negative control, or positive control, which consisted of boric acid) daily. The endpoint that was assessed was survival.

Northern bobwhite quail (*Colinus virginianus*) were administered a nominal limit dose of 105 mg DvSSJ1 dsRNA/kg body weight orally, by gavage, and were observed for 14 d, following OCSPP Guideline 850.2100. In a separate study, and following the same guidelines, *C. virginianus* were administered a nominal limit dose of 2000 mg IPD072Aa protein per kg body weight. A total of 20 birds were used in each study with 5 males and 5 females per treatment (DvSSJ1 dsRNA or the IPD072Aa protein and the negative control water). The endpoints that were assessed included mortality, body weight, abnormal behavior, and signs of toxicity.

Mice *(Mus musculus)* were orally exposed at a limit dose of 2000 mg IPD072Aa protein/kg body weight for 14 d, following OECD, Section 4 (Part 423), with the following exceptions: number of animals (n = 6 males and n = 6 females per treatment), addition of a vehicle control (deionized water) group, and addition of a Bovine Serum Albumin (BSA) protein control group. Male and female (nulliparous and non-pregnant) Crl:CD1(ICR) mice were used in the study. The endpoints that were assessed included mortality, abnormal behavior, and signs of toxicity.

## Statistical Analysis

Statistical analyses of surrogate insect species other than honey bee and quail were conducted using SAS™ software, Version 9.4 (SAS Institute Inc., Cary, NC, USA) separately for each study and each measured endpoint. Statistical analysis of survival data was conducted using Fisher’s exact test (SAS PROC MULTTEST) to determine if the survival observed for the DvSSJ1 dsRNA or the IPD072Aa protein treatments were less than the survival observed with the negative control treatment included in each study. The statistical analysis methods used to assess the weight of surviving insects were dependent upon the validity of statistical assumptions for each data set. For some experiments, the normality assumption was satisfied by the data distributions of the test and control entries, thus a two-sample t-test (SAS PROC TTEST) or an analysis of variance (SAS PROC GLIMMIX) was conducted to assess if the test diet caused growth inhibition. If the normality assumption was not satisfied, SAS PROC NPAR1WAY was used to conduct both Wilcoxon two-sample and Siegel–Tukey test. The Siegel–Tukey test was conducted to further assess for differences in scale between the two treatments^33^ and to determine if exposure to the test diet caused a developmental delay. For days to adult emergence, in all cases the normality assumption was not satisfied; thus, both Wilcoxon two-sample and Siegel–Tukey tests were conducted. For reproduction, a generalized linear mixed model (SAS PROC GLIMMIX) was fit to the reproduction data assuming a Poisson distribution and a fixed effect of treatment. For *F. candida* jar number within each treatment and block were considered random effects. The estimated model was used to test if the reproduction from the adults fed the artificial insect diet containing IPD072Aa protein or DvSSJ1 dsRNA was less than the reproduction from the adults fed the assay control diet. For each of these tests, all *P*-values were considered significant if <0.05.

Statistical analyses of honey bee data were conducted using CETIS Version 1.8^34^ for each study and each measured endpoint. All comparisons for determination of a NOED and LOED were made at ≥95% level of certainty (*p* < .05) and compared on a per replicate (individual well or bee) basis. Statistical analysis of survival or emergence data was conducted using Fisher’s Exact Test with Bonferroni-Holm’s Adjustment. Weight data were first evaluated by conducting Shapiro-Wilk’s Test to assess normality of the distribution and Bartlett’s Test to assess homoscedasticity, and then the results of these tests were used to select the statistical method used in comparisons and determination of NOED and LOED values. For IPD072Aa larval weight data, Dunn’s Test with Bonferroni-Holm’s Adjustment was used for comparisons. For IPD072Aa adult weights, Dunnett’s multiple comparison test was used for comparisons. For DvSSJ1 dsRNA larva and adults, equal variance two-sample t-Tests were used for comparisons of weight.

Statistical analyses of Northern bobwhite quail data were conducted using Mini Tab 17 (Minitab, Inc., College Station, PA) or SAS v9.4. After confirming that statistical assumptions were satisfied, appropriate t-tests were run to determine whether there were differences in weight between sexes and between treatments prior to treatment. Mean measured body weights, calculated body weight change and weekly feed consumption per bird per day were similarly analyzed at the end of the study. Nonparametric data was analyzed using the Wilcoxon’s Rank Sum Test [α = 0.05;.^35, 36^]

The nominal oral limit dose tested in this limit test and corresponding mortality data were used to empirically estimate whether the median lethal dose (LD_50_) and the No Observed Effect Level (NOEL) were greater or less than the highest nominal concentration tested. A similar approach was used to establish the LD_50_ for the mouse study.

## Results and Discussion

*Pollinators and Pollen Feeders*: Based on the worst-case assumption that honey bee larvae consume 2 mg of DP23211 maize pollen during larval development,^22^ the worst-case EECs for the DvSSJ1 dsRNA and the IPD072Aa protein are 4.04 × 10^−3^ ng/larvae and 2.6 ng/larvae dry weight, respectively; Table 2). No adverse effects on honey bee larvae (larval or pupal survival, adult emergence, or adult weight at emergence) were observed and the no observed effect doses (NOEDs) were determined to be 4.0 ng DvSSJ1 dsRNA/larvae (Table 4; Supplemental Table 1) and 200 ng IPD072Aa protein/larvae (Table 5; Supplemental Table 2). The margins of exposure (MOEs) for honey bee larvae exposed to the DvSSJ1 dsRNA or IPD072Aa protein in DP23211 maize pollen are 990X and 77X the worst-case EECs, respectively (Table 4 and Tables 5).

Based on the worst-case assumption that honey bee adults consume 4.3 mg of maize pollen per day,^23^ the worst-case EECs for the DvSSJ1 dsRNA and the IPD072Aa protein are 8.69 × 10^−3^ ng/bee per day and 5.59 ng/bee per day dry weight, respectively; Table 2). No adverse effects on honey bee adults (adult weight and survival) were observed and the no observed effect dietary-doses (NOEDDs) were determined to be 26 ng DvSSJ1 dsRNA/bee/day (Table 4; Supplemental Table 1) and 1,300 ng IPD072Aa protein/bee/day (Table 5; Supplemental Table 2). The MOEs for honey bee adults are 2,993X and 233X the worst-case EECs, respectively (Table 4 and Tables 5).

The worst-case EECs for non-target pollen-feeding Lepidoptera exposed to the DvSSJ1 dsRNA and the IPD072Aa protein in DP23211 maize pollen are 2.02 × 10^−3^ ng/mg and 1.3 ng/mg, respectively (Table 2). The most sensitive target species tested is a useful indicator of potential effects on NTOs.^37^ The LC_50_ of DvSSJ1 dsRNA and the IPD072Aa protein for the most sensitive target pest (*D. virgifera virgifera*) are 0.036 ng/mg and 26 ng/mg, respectively [,^6^ Boeckman et al., in preparation]. For DP23211 maize, the worst-case EECs for non-target lepidopterans are 18–20X below the levels required to elicit 50% mortality in the most sensitive target pest (Table 4 and Table 5). Previously, the lack of activity of DvSSJ1 dsRNA and the IPD072Aa protein on four different families of Lepidoptera was demonstrated as part of a spectrum of activity assessment [^6^ Boeckman et al., in preparation]. Therefore, no additional laboratory bioassays were conducted to assess hazard of the DvSSJ1 dsRNA and the IPD072Aa protein on non-target lepidopterans, and the potential risk to non-target pollen-feeding lepidopterans from exposure to DvSSJ1 dsRNA and the IPD072Aa protein via pollen is considered low. Similar to non-target lepidopterans, the amount of exposure to other pollen-feeding non-target organisms (for example, some coccinellids) is 18–20X below the levels required to elicit 50% mortality in the most sensitive target pest (Table 4 and Tables 5), and the potential risk to non-target pollinators and pollen feeders from exposure to DvSSJ1 dsRNA and the IPD072Aa protein via pollen is considered low.

Refined EECs were not calculated for pollinators and pollen feeders (Table 2), due to the high MOEs using the worst-case assumptions (Table 4 and Table 5). Nevertheless, under a more realistic environmental scenario, several factors would reduce exposure of non-target pollinators and pollen feeders to the DvSSJ1 dsRNA and the IPD072Aa protein in DP23211 maize pollen. For example, honey bees forage over long distances (up to 6–8 miles) to collect pollen and nectar.^38,39^ Adult honey bees are likely to feed on pollen from a variety of different plant species,^40,41^ and therefore maize pollen, specifically DP23211 maize pollen, is unlikely to be the only food consumed. Similarly, most non-target pollen-feeding Lepidoptera larvae do not feed on pollen directly but are indirectly exposed to pollen as they feed on host plants. For example, Monarch butterfly larvae (*Danaus plexippus*, Lepidoptera: Nymphalidae) use milkweed as their host plant.^42^ Weed management practices may decrease host plant density within maize field and field margins,^43^ and pollen is known to be deposited in close proximity to maize field margins.^44^ The duration of pollen shed for cultivated maize can be variable, but typically, pollen shed lasts between 2 and 14 d and is highly dependent on the maize hybrid, developmental stage, weather, and location.^45^ In the U.S., pollen is shed over 2 weeks between mid-July and mid-August.^42^ Therefore, these worst-case assumptions are extremely conservative, and the potential risk of cultivation of DP23211 maize on pollinators and pollen feeders is considered negligible.

*Soil Dwelling Detritivores*: Soil-dwelling detritivores are most likely to consume senescent maize tissues that are incorporated into the soil post-harvest.^5^ Therefore, for soil dwelling detritivores, the route of exposure to the DvSSJ1 dsRNA and the IPD072Aa protein is via ingestion of senescent DP23211 maize tissues, and the worst-case EECs are 2.99 × 10^−2^ ng/mg and 24 ng IPD072Aa protein/mg, respectively (Table 2). For DvSSJ1 dsRNA, no adverse effects on springtail reproduction or survival were observed, and the no observed effect concentration (NOEC) was determined to be 1 ng DvSSJ1 dsRNA/mg diet (Table 4 and Supplemental Table 1). For IPD072Aa, no adverse effects on springtail survival were observed, and the NOEC was determined to be 500 ng IPD072Aa/mg (Table 5; Supplemental Table 2). A statistically significant difference was observed for the sub-lethal endpoint reproduction (mean number of offspring per jar), however this was not biologically meaningful based on acceptability guidelines for collembolan reproduction established by OECD, as well as the overlapping range of offspring observed across treatments (Table 5; Supplemental Table 2). The MOEs for springtail exposed to the DvSSJ1 dsRNA or IPD072Aa protein in senescent DP23211 maize tissue are 33X and 21X the worst-case EECs, respectively (Table 4 and Table 5). These worst-case assumptions are conservative and, based on the high MOEs and narrow spectrum of activity, refined EECs were not calculated (Table 2) and the risk of cultivation of DP23211 maize to soil dwelling detritivores is considered negligible.

*Aquatic detritivores*: The DvSSJ1 dsRNA and the IPD072Aa protein DP23211 maize tissues could enter the water column and present a potential route of exposure to non-target aquatic detritivores. Maize tissue has previously been identified as a potential route of exposure for aquatic detritivores.^13^ Although aquatic habitats may be located near agricultural areas, exposure of aquatic detritivores to GM crops is limited temporally and spatially,^5^ and aquatic exposure to Bt corn has been shown to be extremely low.^46^ The EPA standard agricultural field-farm pond model (also called the US EPA standard pond model^47^) has been used to estimate pesticide runoff and has been adapted to model exposure of aquatic NTOs to newly expressed proteins in GM crops.^16,24,48^ The worst-case EECs for DvSSJ1 dsRNA and the IPD072Aa protein are 2.46 × 10^–22^ mg/l and 0.124 mg/l, respectively (Table 2). The assumptions used for the worst-case EECs are conservative because it is very unlikely that all above ground maize tissue from a 10-ha field will enter a 1-ha pond and it is unlikely that all of the protein in maize tissue will enter the water or remain active. Therefore, based on negligible potential for exposure, tier I hazard studies on non-target aquatic organisms do not inform the ERA for DP23211 maize. As discussed previously, the LC_50_ of DvSSJ1 dsRNA and the IPD072Aa protein for the most sensitive target pest (*D. virgifera virgifera*) are 0.036 ng/mg and 26 ng/mg, respectively, [^6^ Boeckman et al., in preparation], which are 146X and 210X the worst-case EECs, respectively (Table 4 and Tables 5). Therefore, the risk of cultivation of DP23211 maize to aquatic detritivores is considered negligible.

*Herbivores, Predators, and Parasitoids*: Insect herbivores are typically considered agricultural pests (for example, aphids, thrips, etc.), but some beneficial non-target organisms may incidentally eat maize tissue or supplement their diets with maize tissue when needed [for example, some coccinellid species;.^25^] The worst-case EEC for non-target herbivores (exposed to the maximum concentration of DvSSJ1 dsRNA or the IPD072Aa protein in above-ground DP23211 maize tissue) is 0.113 ng/mg and 39 ng/mg, respectively (Table 2). A refined EEC for non-target herbivores (exposed to the mean concentration of DvSSJ1 dsRNA or the IPD072Aa protein in above-ground DP23211 maize tissue) is 0.0646 ng/mg and 16 ng/mg, respectively (Table 2).

For insect predators or parasitoids, the route of exposure to the DvSSJ1 dsRNA and the IPD072Aa protein is via consumption of prey that has previously consumed tissue from a DP23211 maize plant (Table 2). Because a predator typically does not feed directly on large amounts of the maize plant, one factor to consider in the exposure assessment for predators or parasitoids is the amount of DvSSJ1 dsRNA or the IPD072Aa protein that transfers and accumulates in the prey. Secondary exposures via prey are influenced not only by the rates of ingestion, digestion and excretion of plant material by the prey [see,^39^for review], but also are affected by the stability of the DvSSJ1 dsRNA or the IPD072Aa protein within the prey. The EECs for predators and parasitoids were based on the conservative assumption that there is no degradation of the DvSSJ1 dsRNA or the IPD072Aa protein in prey (i.e., 100% of the DvSSJ1 dsRNA or the IPD072Aa protein is transferred from plant to prey to predator). The worst-case and refined EECs for predators and parasitoids are therefore the same as the worst-case and refined EECs for herbivores (Table 2); however, environmental exposure through the predator and parasitoid pathway is likely much lower. For example, previously an assumption of 20% transfer from plant to prey to predator has been used to calculate EECs.^48^

Five surrogate species representing the predator and parasitoid functional group were assessed: one Neuroptera (*C. rufilabris*), one Hymenoptera (*P. foveolatus*), and three Coleoptera, which included one Staphylinidae (*Dalotia coriaria*), and two Coccinellidae (*C. maculata* and *H. convergens*). No adverse effects on *C. rufilabris* survival or pupation were observed, and the NOECs were determined to be 1 ng DvSSJ1 dsRNA/mg diet (Table 4; Supplemental Table 1) and 500 ng IPD072Aa protein/mg diet (Table 5; Supplemental Table 2). No adverse effects on *P. foveolatus* survival were observed, and the NOECs were determined to be 1 µg DvSSJ1 dsRNA/ml (Table 4; Supplemental Table 1) and 1000 µg IPD072Aa protein/ml diet (Table 5; Supplemental Table 2). The worst-case and refined MOEs for these representative Neuroptera and Hymenoptera species exposed to the DvSSJ1 dsRNA via prey are both 9X and 15X, respectively (Table 4). The worst-case MOEs for *C. rufilabris* and *P. foveolatus* exposed to the IPD072Aa protein are 13X and 26X, respectively (Table 5), and the refined EEC are 31X and 63X, respectively (Table 5). The overall MOE values for these surrogate species representing the predator and parasitoid functional group indicate that the DvSSJ1 dsRNA and the IPD072Aa protein in DP23211 maize is unlikely to be harmful at environmentally realistic concentrations.

Based on the specificity of the DvSSJ1 dsRNA (Boeckman et al., in preparation), non-target Coleoptera are more likely to be sensitive than NTOs from other orders. No adverse effects on *D. coriaria* survival, or *C. maculata* or *H. convergens* survival, weight, or number of days to adult emergence were observed, and the NOECs were determined to be 1 ng DvSSJ1 dsRNA/mg diet (Table 4; Supplemental Table 1). The worst-case and refined MOEs for these non-target Coleoptera (from the families Staphylinidae and Coccinellidae) exposed to the DvSSJ1 dsRNA via prey or direct feeding on DP23211 maize tissues are 9X and 15X, respectively (Table 4), and based on these MOEs, DvSSJ1 dsRNA in DP23211 maize is unlikely to be harmful at environmentally realistic concentrations.

Based on the spectrum of activity of the IPD072Aa protein being within the order Coleoptera,^6^ non-target coleopterans are more likely to be sensitive than NTOs from other orders. No adverse effects on *D. coriaria* survival were observed, and the NOECs were determined to be 1000 µg IPD072Aa protein/ml diet (Table 5; Supplemental Table 2). The worst-case and refined MOEs for *D. coriaria* exposed to the IPD072Aa protein via prey or direct feeding on DP23211 maize tissues are 26X and 63X, respectively (Table 5), and based on these MOEs, DP23211 maize is unlikely to be harmful at environmentally realistic concentrations.

At the highest concentrations tested (500 ng/mg and 1000 ng/mg), *C. maculata*, had increased mortality, and significant effects were observed on sub-lethal endpoints (weight, or number of days to adult emergence; Supplemental Table 2). *C. maculata* survival, weight, and number of days to adult emergence were not significantly different than negative control in the 100 ng/mg treatment (Table 5, Supplemental Table 2). Therefore, the survival NOEC is 100 ng/mg, which results in worst-case and refined MOEs of 3X and 6X. Similarly, at the highest concentration tested (1000 ng/mg), *H. convergens* had significantly increased mortality, significantly lower weight and a higher number of days to adult emergence compared with the control (Supplemental Table 2). At 500 ng/mg, *H. convergens* also had significantly lower weight and a higher number of days to adult emergence (Table 5, Supplemental Table 2), but survival was not significantly different. The survival NOEC for *H. convergens* is 500 ng/mg, which correspond to MOEs of 13X and 31X the worst-case and refined EECs (Table 5; Supplemental Table 2).

Some sublethal effects were observed in non-target coccinellids, and the survival NOEC for *C. maculata* is less than the 10X threshold, which is routinely used to gauge the need for either additional hazard or exposure assessment. However, in the highest treatment tested (1000 ng/mg diet, which represents 25X the worst-case EEC and 62.5X the refined-EEC), the mortality of *C. maculata* was only 36.7%, which is well below the 50% effect threshold recommended for triggering additional hazard testing (Supplemental Table 2). As previously discussed, a less than 50% effect observed at a maximum hazard dose (i.e., 10X the EEC) indicates minimal risk.^39^ Furthermore, at the highest tested concentration, the sub-lethal endpoints also did not approach the 50% effect threshold. Mean adult weight in the 1000 ng IPD072Aa/mg treatment was 10.4 mg, compared to 12.0 mg in the control treatment (Supplemental Table 2), and median number of days to adult emergence was 16 d in the 1000 ng IPD072Aa/mg treatment, compared to 14 d in the control treatment (Supplemental Table 2). Similarly, for *H. convergens* mortality in the 1000 ng IPD072Aa/mg treatment was 56.7%. *H. convergens* mean adult weight in the 1000 ng IPD072Aa/mg treatment was 8.63 mg, compared to 19.7 mg in the control treatment (Supplemental Table 2), and median number of days to adult emergence was 22 d in the 1000 ng IPD072Aa/mg treatment, compared to 14 d in the control treatment (Supplemental Table 2). These effects observed in the 1000 ng IPD072Aa/mg treatment (which represents 25X the worst-case EEC and 62.5X the refined-EEC) approach or cross the 50% threshold, which triggered further consideration of exposure.

Several factors are likely to reduce the actual exposure of non-target coccinellids to the IPD072Aa protein via prey or direct feeding on DP23211 maize tissues, below the worst-case EECs and the refined EECs used in this screening assessment. For example, for predators, we used the assumption that there is no degradation of the protein in prey, and 100% of the protein transfers from DP23211 maize to prey to predator. In reality, it is likely that there is some protein degradation and/or clearance of the protein in prey, and the amount of protein transferred to predator is overestimated by these assumptions. Additionally, not all of the prey that these predators consume may feed exclusively on DP23211 maize tissue. Furthermore, coccinellids consume a variety of foods including pollen (which has low concentrations of the IPD072Aa protein) or prey that fed on other plant tissues so the assumption that a population of predators will consume only DP23211 maize tissue or prey that consumed DP23211 maize tissue is highly conservative. Therefore, based on these mitigating factors and the established MOEs, there are unlikely to be biologically relevant adverse effects on populations of non-target Coccinellids due to cultivation of DP23211 maize under realistic biological conditions.

*Insectivorous Birds*: Similar to insect predators, the worst-case EECs for insectivorous birds is based on the highest maximum concentration of the DvSSJ1 dsRNA and the IPD072Aa protein in above ground tissue (0.113 ng/mg and 39 ng/mg, respectively; Table 2). The refined EEC was calculated using the highest mean concentration of the IPD072Aa protein (0.0646 ng/mg and 16 ng/mg, respectively; Table 2). No adverse effects on survival, weight, behavior, or signs of toxicity were observed in *Colinus virginianus* fed diet containing 105 mg DvSSJ1 dsRNA/kg body weight (Table 4; Supplemental Table 1) or a diet containing 2000 mg IPD072Aa protein/kg body weight (Table 5; Supplemental Table 2). The MOEs for *C. virginianus* are 929X and 51X the worst-case EECs for the DvSSJ1 dsRNA and the IPD072Aa protein, respectively (Table 4 and Table 5), and the MOEs are much higher if the refined EECs are considered (Table 4 and Table 5). The same mitigating factors previously discussed for insect predators also would apply to the exposure assessment for insectivorous birds (food and prey choice, prey consumption of DP23211 maize, etc.); therefore, there are unlikely to be biologically relevant adverse effects on populations of insectivorous birds due to cultivation of DP23211 maize.

*Granivorous Mammals*: Using assumptions about the amount of maize grain in diet, and calculating the dietary daily dose for wild mammals, the worst-case EECs for the DvSSJ1 dsRNA and the IPD072Aa protein are 0.0026 mg/kg body weight and 1.156 mg/kg body weight, respectively (Table 2). A Tier I hazard study on a surrogate wild mammal species was not conducted based on negligible potential for exposure to the DvSSJ1 dsRNA. As summarized in more detailed reviews, nucleic acids are present in human and animal food and feed, their consumption has not been associated with adverse health effects,^37,49,50^ and endogenous RNAi is known to occur in plants and animals, including those used as food and feed [Ambros.^51–57,63^] The physical, chemical, enzymatic, and molecular barriers to exposure and activity of dietary dsRNAs ingested by humans and other mammals have been well described in the context of agricultural biotechnology and the safety assessment of crops containing RNAi technology.^49,58–60,61,62^ These barriers to exposure and activity of ingested dsRNAs are anticipated to prevent or significantly reduce human and animal exposure to DvSSJ1 dsRNA from consumption of foods or feed containing DP23211 maize. Therefore, based on the lack of exposure, there are unlikely to be adverse effects on populations of wild mammals due to cultivation of DP23211 maize.

For the IPD072Aa protein, a study using the limit dose of 2000 mg IPD072Aa protein/kg body weight, and no mortality or evidence of acute oral toxicity (based on evaluation of body weight, clinical signs, and gross pathology) was observed in *Mus musculus* [Table 5; Supplemental Table 2;.61] The MOE for *M. musculus* is 1730X the worst-case EEC (Table 5), and refined EECs were not calculated due to high MOEs using worst-case assumptions. There are several factors that would reduce exposure to granivorous mammals under more realistic environmental conditions, and there are therefore unlikely to be adverse effects on populations of non-target mammals due to cultivation of DP23211 maize.

## Conclusions

In conclusion, no adverse effects on NTO populations are expected as a result of cultivation of DP23211 maize based on the levels of exposure to DvSSJ1 dsRNA and the IPD072Aa protein and the results of Tier I laboratory toxicity studies. The approach used to assess environmental risk followed the EPAs risk assessment framework,^10^ which is robust and suitable for assessing risk of non-Bt proteins^11^ and dsRNA.^5,12^ The spectrum of activity of DvSSJ1 dsRNA (Boeckman et al., in preparation) and the IPD072Aa protein,^6^ as well as information about the mode of action^7,8^ were considered as part of problem formulation. Ingestion of DvSSJ1 dsRNA and the IPD072Aa protein by the target pest, WCR, primarily causes insect mortality. Therefore, mortality was used as the primary endpoint from Tier 1 studies for calculating MOEs. Sub-lethal endpoints were also assessed for several of the non-target organisms as part of the characterization of the product. For DvSSJ1 dsRNA, no adverse effects on NTO sublethal endpoints were observed, and DvSSJ1 dsRNA in DP23211 maize is unlikely to be harmful at environmentally realistic concentrations. For the IPD072Aa protein, a statistically significant difference was observed for springtail reproduction; however, this was unlikely to be biologically relevant. With the exception of two Coccinellids, *C. maculata* and *H. convergens*, no other statistically significant differences in survival or sublethal endpoints assessed for other NTOs were observed and the MOEs were >10X. For *C. maculata* and *H. convergens*, further refinement of the exposure assessment was triggered, based on MOEs <10X the worst-case EECs. The magnitude of the observed differences in the highest treatment tested (1000 ng IPD072Aa/mg treatment, which represents 25X the worst-case EEC and 62.5X the refined-EEC) was compared to 50% effect threshold, to assess potential for population level risk. After considering all of the mitigating factors that would reduce the actual exposure of non-target Coccinellids to the IPD072Aa protein in DP23211 maize (including prey choice, proportion of the population potential consuming DP23211 maize, protein stability in prey, etc.), risk to non-target Coccinellid populations is expected to be negligible.

## Supplementary Material

Supplemental Material
