# Supplementary material for: Environmental risk assessment of the DvSSJ1 dsRNA and the IPD072Aa protein to non-target organisms
Source: GM Crops Food. 2021 Dec 14;12(1):459–78. doi: 10.1080/21645698.2021.1982348 (PMC8820247; doi:10.1080/21645698.2021.1982348)
Supplement: Supplemental Material [file KGMC_A_1982348_SM2781.docx]

**Environmental Risk Assessment of the DvSSJ1 dsRNA and the IPD072Aa Protein to Non-target Organisms**

Chad J. Boeckman*, Jennifer A. Anderson, Christopher Linderblood, Taylor Olson, Jason Roper, Kristine Sturtz, Carl Walker; Rachel Woods

Corteva Agriscience™, 7300 NW 62nd Avenue, Johnston, IA 50131, USA

*Corresponding Author

Chad Boeckman

Corteva Agriscience™

Johnston, IA 50131, USA

[Chad.Boeckman@corteva.com](mailto:Chad.Boeckman@corteva.com)

(515) 535-6160 (phone)

**Supplemental Methods:**

*DvSSJ1 210bp Characterization:* DvSSJ1 210 bp dsRNA was produced using an *in-vitro* transcript and a proprietary method by a third party. The DvSSJ1 210bp purified test substance was suspended in water and shipped to the test site. Upon arrival at the test site DvSSJ1 210 bp dsRNA was aliquoted and stored in a -80°C freezer until use. Prior to use in study, the concentration of the DvSSJ1 210 bp dsRNA was determined using multiple agarose gel electrophoresis-based densitometry evaluations. Serial dilutions of a DNA standard (208 bp Nucleosome Control DNA, New England Biolabs) were used as concentration standards for densitometry analysis. Each analysis included a 1% agarose gel containing a set of concentration standards and four dilutions of DvSSJ1 210bp dsRNA. Gel images were captured and the densitometric value of each band was measured by Quantity One® 1-D analysis software. Non-linear regression analysis (logistic power regression) was used to create a standard curve from the DNA standards and the equation was then used to calculate the concentration for each DvSSJ1 210 bp dsRNA sample, with final results adjusted by the associated dilution. Three separate analysts conducted independent assessments and the three values were averaged to arrive at a final concentration of 2.1 mg DvSSJ1 /mL.

Purity was also determined for the DvSSJ1 210bp dsRNA sample using high-resolution polyacrylamide gel electrophoresis (PAGE). Three separate analysts conducted three fully independent evaluations which were averaged to arrive at a final purity of 95.1%. Sequence identity was verified using reverse transcription-PCR and Sanger-based sequencing. Sanger-based sequencing was conducted in both forward and reverse directions to cover every nucleotide using M13 forward and reverse primers. Sequencher Version 4.8 Software (Gene Codes Corporation, Ann Arbor, MI) was used to analyze and assemble the sequences. Equivalency to the DP23211 maize derived DvSSJ1 dsRNA was established by isolating dsRNA from DP23211 maize tissues, followed by confirming sequence identity (via reverse transcription into cDNA and PCR amplification) and through the use of Northern blots confirming band presence at the expected gel locations. Finally, biological activity for the DvSSJ1 dsRNA was confirmed using western corn rootworm.

*QuantiGene Analysis:* Total RNA was extracted by adding 500 µl of RNA lysis buffer (50 mM sodium citrate pH 4.5, 25 mM EDTA, 75 mM sodium chloride, 1% sodium dodecyl sulfate, and 1% β-mercaptoethanol [freshly added]) to pre-weighed tissue samples. An equal volume (500 µl) of acid-phenol:chloroform mixture (1:1) was added to the slurry and samples were ground with 1.0 mm zirconium oxide beads. After grinding, samples were incubated for 5 minutes at approximately 65 °C then centrifuged in Phase Lock Gel-Heavy tubes to separate the phases. The aqueous phase was removed, transferred to fresh tubes, and 1.5 ml TRI-reagent and 2 ml 200-Proof Ethanol were added. The total RNA was purified using Zymo Direct-zol RNA Miniprep Plus Kit and eluted from the column in 100 µl of RNase-free water. The concentration of total RNA was determined using a NanoDrop 2000 UV-Vis Spectrophotometer. Representative samples were checked for RNA quality and integrity by gel electrophoresis. Samples that showed poor RNA quality were excluded from DvSSJ1 dsRNA concentration analysis. Total RNA was stored frozen (-80 °C freezer unit) until QuantiGene analysis.

QuantiGene analysis was used to determine the concentration of DvSSJ1 dsRNA in tissues derived from DP23211 maize. The QuantiGene method was internally validated to demonstrate method suitability. The DvSSJ1 QuantiGene Plex Assay method utilized magnetic beads specific to the target to measure the amount of DvSSJ1 dsRNA in samples. Prior to analysis, total RNA was diluted to 10-30 ng total RNA/µl (equal to 200 – 600 ng total RNA/well) in QuantiGene Homogenizing Solution (QHS). Standards and samples (both typically analyzed in triplicate wells) were first denatured (95 °C for five minutes) and annealed (55 °C) in a 96‐well PCR plate with a sequence‐specific probe set designed by Affymetrix that included Capture Extenders (CE), Label Extenders (LE), and Blocking Probes. The mixture of sample and probe set was transferred to a hybridization plate containing bead mix (100 µl total volume) for overnight hybridization at approximately 54 °C. Following hybridization, a magnetic separation device was used to wash unbound substances from the plate. Signal was amplified on the LE probes by sequential incubations (each 1 – 2 hours at approximately 50 °C) with pre‑amplifier, amplifier, and label probes, with each incubation followed by a wash step to remove unbound substances. Each well was then incubated with the fluorescent protein streptavidin phycoerythrin (SAPE; 30 minutes at room temperature) and then washed. The SAPE generated a signal that was proportional to the amount of DvSSJ1 dsRNA present in the reaction. The median fluorescence intensity (MFI) of each well was then determined using a MAGPIX Multiplex Reader running xPonent v4.2 software.

*IPD072Aa Protein Concentration and Characterization:* The concentration of IPD072Aa protein was determined using a quantitative enzyme‑linked immunosorbent assay (ELISA) method that was internally validated to demonstrate method suitability. Processed tissue sub‑samples were weighed at the following target weights: 5 mg for pollen; 10 mg for leaf; 20 mg for grain and root; and 30 mg for forage. Leaf, pollen, whole plant, forage, and grain samples were extracted with 0.60 ml of chilled 25% StabilZyme Select in phosphate‑buffered saline containing polysorbate 20 (PBST), and root samples were extracted in chilled H5 buffer (comprised of 90 mM HEPES, 140 mM sodium chloride, 1.0% polyethylene glycol, 1.0% PVP‑40, 1.0% bovine serum albumin, 0.007% thimerosal, and 0.3% polysorbate 20). All extracted samples were centrifuged, and then supernatants were removed and prepared for analysis.

Prior to analysis, samples were diluted as applicable with 25% StabilZyme Select in PBST. Standards (typically analyzed in triplicate wells) and diluted samples (typically analyzed in duplicate wells) were incubated in a plate pre-coated with an IPD072Aa specific antibody. Following incubation, unbound substances were washed from the plate and the bound IPD072Aa protein was incubated with a different IPD072Aa specific antibody conjugated to the enzyme horseradish peroxidase (HRP). Unbound substances were washed from the plate. Detection of the bound IPD072Aa antibody complex was accomplished by the addition of substrate, which generated a colored product in the presence of HRP. The reaction was stopped with an acid solution and the optical density (OD) of each well was determined using a plate reader.

To produce the IPD072Aa protein it was expressed in an *E. coli* strain BL21(DE3) through fermentation as a fusion protein with an N-terminal His tag and a Factor Xa cleavage site. The protein expression was controlled under an inducible promoter and the induction was done with 1mM IPTG at 16°C. The His-tagged protein was purified from the *E. coli* cell lysates using Ni-NTA affinity chromatography. The fusion tag was then cleaved by immobilized trypsin and removed by further Ni-NTA affinity chromatography followed by additional purification with Q Sepharose-based anion exchange column chromatography. Tangential flow filtration was used to exchange the buffer to 50 mM ammonium bicarbonate. The protein was then lyophilized and stored at −80°C. The concentration of the lyophilized protein was determined by amino acid composition analysis and showed 0.80 mg protein/mg

lyophilized powder. The purity was analyzed using sodium dodecyl sulfate polyacrylamide gel electrophoresis, which revealed >95%

purity on a total protein basis. To establish equivalency with the IPD072Aa protein expressed in DP23211 maize, additional characterization of the protein was conducted using western blotting, N-terminal sequencing, mass spectrometry for intact mass determination and peptide mapping, glycosylation staining, and insecticidal activity using WCR.

**Supplement Table 1**. **Early Tier Laboratory Study Results for the DvSSJ1 dsRNA on Representative Non-Target Organisms**

| **Species** | **Concentration of DvSSJ1 dsRNA** | **Mortality (%)**  **(P-value)** | **Sublethal endpoint A**  **(P-value)** | **Sublethal endpoint B**  **(P-value)** |
| --- | --- | --- | --- | --- |
| *Apis mellifera*  (Honey bee larvae) ^1^ | 0 (ng/larvae) | 0% (larva1) 28% (pupal) | 72% | 100.3 mg |
|  | 4.0 (ng/larvae) | 3% (larval)  (0.500)  37% (pupal)  (0.2780) | 61% | 106.0 mg  (0.9060) |
| *Apis mellifera*  (Honey bee adult) ^2^ | 0 (ng/bee/day) | 10% | 104.7 mg |  |
|  | 26 (ng/bee/day) | 10%  (1.000) | 100.7 mg  (0.0975) |  |
| *Folsomia candida* (Springtail) ^3^ | 0 (ng /mg diet) | 3.75% | 319 (# offspring) | 268 – 399 (range) |
|  | 1 (ng /mg diet) | 0%  (1.000) | 345 (# offspring)  (0.8290) | 264 – 462 (range) |
| *Chrysoperla rufilabris*  (Green lacewing) ^4^ | 0 (ng /mg diet) | 2.56% | 100% |  |
|  | 1 (ng /mg diet) | 2.5%  (0.7595) | 100%  (NA) |  |
| *Coleomegilla maculata*  (Pink spotted lady beetle)^5^ | 0 (ng /mg diet) | 0% | 12.6 mg | 15 days |
|  | 1 (ng /mg diet) | 10.3%  (0.1124) | 13.0 mg  (0.7611) | 14 days  (0.9429) |
| *Hippodamia convergens*  (Convergent ladybird beetle) ^6^ | 0 (ng /mg diet) | 6.67% | 19.1 mg | 15 days |
|  | 1 (ng /mg diet) | 7.41%  (0.6531) | 18.0 mg  (0.1157) | 15 days  (0.3972) |
| *Dalotia coriari*  (Rove beetle) ^7^ | 0 (ng /mg diet) | 3.33% |  |  |
|  | 1 (ng /mg diet) | 0%  (1.0000) |  |  |
| *Pediobius foveolatus*  (parasitic hymenoptera) ^8^ | 0 (ng /ml diet) | 17.2% |  |  |
|  | 1 (ng /ml diet) | 6.67%  (0.9537) |  |  |
| *Colinus virginianus*  (Northern bobwhite quail) ^9^ | 0 (mg/kg bw) | 0% | 200.0 g | 200.2 g |
|  | 105 (mg/kg bw) | 0%  (NA) | 208.0 g  (0.1981) | 206.5 g  (0.2510) |

^1^ For honey bee larvae, mortality was assessed for larval and pupal stages; the sublethal endpoint A was cumulative percent adult emergence (%); Sublethal endpoint B was mean adult weight at emergence (mg) N = 36 organisms per treatment at test initiation.

^2^ For honey bee adults, sublethal endpoint A was adult weight (mg); N = 30 organisms per treatment at test initiation.

^3^ For springtail, sublethal endpoint A was reproduction (mean number of offspring per jar); Range of offspring per jar is presented in Sublethal endpoint B column. N = 80 organisms per treatment at test initiation.

^4^ For green lacewing, sublethal endpoint A was pupation rate (%); P-Value was not calculated (NA); N = 40 organisms per treatment at test initiation.

^5^ For pink spotted lady beetle, sublethal endpoint A was media adult weight (mg); sublethal endpoint B was median number of days to adult emergence (days); N = 30 organisms per treatment at test initiation.

^6^ For convergent ladybird beetle, sublethal endpoint A was mean adult weight (mg); Sublethal endpoint B was median number of days to adult emergence (days). N = 30 organisms per treatment at test initiation.

^7^ For rove beetle, no sublethal endpoints were assessed. N = 30 organisms per treatment at test initiation.

^8^ For *Pediobius foveolatus*, no sublethal endpoints were assessed. N = 30 organisms per treatment at test initiation.

^9^ For northern bobwhite quail, sublethal endpoint A was mean body weight (g) recorded on day 7; sublethal endpoint B was mean body weight (g) recorded on day 14 post-dosing; additional endpoints included food consumption and changes in behavior. N = 10 males and 10 females per treatment.

**Supplement Table 2**. **Early Tier Laboratory Study Results for the IPD072Aa Protein on Representative Non-Target Organisms**

| **Species** | **Concentration of IPD072Aa protein** | **Mortality (%)**  **(P-value)** | **Sublethal endpoint A**  **(P-value)** | **Sublethal endpoint B**  **(P-value)** |
| --- | --- | --- | --- | --- |
| *Apis mellifera*  (Honey bee larvae) ^1^ | 0 (ng/larvae) | 8% (larval)  18% (pupal) | 75% | 108.1 mg |
|  | 100 (ng/larvae) | 14% (larval)  (0.7101)  6% (pupal)  (1.000) | 81%  (1.000) | 109.5 mg  (0.8233) |
|  | 200 (ng/larvae) | 8% (larval)  (0.6631)  6% (pupal)  (1.000) | 86%  (1.000) | 106.8 mg  (0.5798) |
| *Apis mellifera*  (Honey bee adult) ^2^ | 0 (ng/bee/day) | 3% | 104.2 mg |  |
|  | 640 (ng/bee/day) | 10%  (0.6120) | 99.37 mg  (0.2621) |  |
|  | 1300 (ng/bee/day) | 10%  (0.6120) | 109.9 mg  (0.9487) |  |
| *Folsomia candida* (Springtail) ^3^ | 0 ng/mg | 0% | 460 (# offspring) | 356 – 571 (range) |
|  | 500 ng/mg | 2.5%  (0.2453) | **394** (# offspring)  **(0.0344)** | 317 – 554 (range) |
| *Chrysoperla rufilabris*  (Green lacewing) ^4^ | 0 ng/mg | 12.5% | 100 %  (NA) |  |
|  | 500 ng/mg | 10.3%  (0.7465) | 100%  (NA) |  |
| *Coleomegilla maculata*  (Pink spotted lady beetle)^5^ | 0 ng/mg | 6.90% | 12.0 mg | 14 days |
|  | 100 ng/mg | 6.67%  (0.7070) | 12.1 mg  (0.5424) | 15 days  (0.1529) |
|  | 500 ng/mg | **26.7%**  **(0.0449)** | **10.7 mg**  **(0.0073)** | **16 days**  **(0.0015)** |
|  | 1000 ng/mg | **36.7%**  **(0.0061)** | **10.4 mg**  **(0.0014)** | **16 days**  **(0.0088)** |
| *Hippodamia convergens*  (Convergent ladybird beetle) ^6^ | 0 ng/mg | 0% | 19.7 mg | 14 days |
|  | 100 ng/mg | 0% | **18.6 mg**  **(0.0236)** | 14 days  (0.0551) |
|  | 500 ng/mg | 0% | **11.3 mg**  **(<0.0001)** | **17 days**  **(<0.0001)** |
|  | 1000 ng/mg | **56.7%**  **(<0.0001)** | **8.63 mg**  **(<0.0001)** | **22 days**  **(<0.0001)** |
| *Dalotia coriari*  (Rove beetle) ^7^ | 0 | 0% |  |  |
|  | 100 | 3.33%  (0.5000) |  |  |
|  | 500 | 10.0%  (0.1186) |  |  |
|  | 1000 | 6.76%  (0.2458) |  |  |
| *Pediobius foveolatus*  (Parasitic hymenoptera) ^8^ | 0 µg/ml | 3.33% |  |  |
|  | 100 µg/ml | 17.2%  (0.0896) |  |  |
|  | 500 µg/ml | 16.7%  (0.973) |  |  |
|  | 1000 µg/ml | 20.0%  (0.0514) |  |  |
| *Colinus virginianus*  (Northern bobwhite quail) ^9^ | 0 (mg/kg bw) | 0 % | 191.04 g | 193.05 g |
|  | 2000 (mg/kg bw) | 0 %  (NA) | 192.30 g  (0.789) | 194.6 g  (0.745) |
| *Mus musculus*  (Mouse) ^10^ | 0 (mg/kg bw) | 0% | 32.4 g (male)  27.2 g (female) | 4.6 g (male)  3.2 g (female) |
|  | 2000 (mg/kg bw) | 0%  (NA) | 33.9 g (male)  26.5 g (female) | 4.8 g (male) 2.6 g (female) |

^1^ For honey bee larvae, mortality was assessed for larval and pupal stages; Sublethal endpoint A was cumulative percent adult emergence (%); Sublethal endpoint B was mean adult weight at emergence (mg). N = 36 organisms per treatment at test initiation.

^2^ For honey bee adults, sublethal endpoint A was adult weight (mg). N = 30 organisms per treatment at test initiation.

^3^ For springtail, sublethal endpoint A was reproduction (mean number of offspring per jar); Bold text designates statistically significant difference (P-value <0.05) was observed. In this case, the statistically difference was determined to be not biologically relevant based on acceptability guidelines for collembolan reproduction established by OECD, as well as the overlapping range of offspring (presented in Sublethal endpoint B column). N = 80 organisms per treatment at test initiation.

^4^ For green lacewing, sublethal endpoint A was pupation rate (%); P-Value was not calculated (NA). N = a target of 40 larvae per treatment at test initiation.

^5^ For pink spotted lady beetles, sublethal endpoint A was mean adult weight (mg); Sublethal endpoint B was median number of days to adult emergence (days); Bold text designates that a statistically significant difference (P-value < 0.05) was observed in this treatment compared to control treatment. N = 30 organisms per treatment at test initiation.

^6^ For convergent ladybird beetle, sublethal endpoint A was mean adult weight (mg); Sublethal endpoint B was median number of days to adult emergence (days); Bold text designates that a statistically significant difference (P-value < 0.05) was observed in this treatment compared to control treatment. N = 30 organisms per treatment at test initiation.

^7^ For rove beetle, no sublethal endpoints were assessed. N = 30 organisms per treatment at test initiation.

^8^ For *Pediobius foveolatus,* no sublethal endpoints were assessed. N = 30 organisms per treatment at test initiation.

^9^ For northern bobwhite quail, sublethal endpoint A was mean body weight (g) recorded on day 7; sublethal endpoint B was mean body weight (g) recorded on day 14 post-dosing; additional endpoints included food consumption and changes in behavior. N = 10 males and 10 females per treatment.

^10^ For mouse, sublethal endpoint A was mean body weight for male mice and female mice (g); Sublethal endpoint B was body weight gain for male mice and female mice (g); P-Values were not calculated. N = 6 males and 6 females per treatment.
